# The Sheep Grimace Scale as an indicator of post-operative distress and pain in laboratory sheep

**DOI:** 10.1371/journal.pone.0175839

**Published:** 2017-04-19

**Authors:** C. Häger, S. Biernot, M. Buettner, S. Glage, L. M. Keubler, N. Held, E. M. Bleich, K. Otto, C. W. Müller, S. Decker, S. R. Talbot, A. Bleich

**Affiliations:** 1 Institute for Laboratory Animal Science and Central Animal Facility, Hannover Medical School, Hannover, Germany; 2 Trauma Department, Hannover Medical School, Hannover, Germany; Universidade do Porto Instituto de Biologia Molecular e Celular, PORTUGAL

## Abstract

The EU Directive 2010/63/EU changed the requirements regarding the use of laboratory animals and raised important issues related to assessing the severity of all procedures undertaken on laboratory animals. However, quantifiable parameters to assess severity are rare, and improved assessment strategies need to be developed. Hence, a Sheep Grimace Scale (SGS) was herein established by observing and interpreting sheep facial expressions as a consequence of pain and distress following unilateral tibia osteotomy. The animals were clinically investigated and scored five days before surgery and at 1, 3, 7, 10, 14 and 17 days afterwards. Additionally, cortisol levels in the saliva of the sheep were determined at the respective time points. For the SGS, video recording was performed, and pictures of the sheep were randomized and scored by blinded observers. Osteotomy in sheep resulted in an increased clinical severity score from days 1 to 17 post-surgery and elevated salivary cortisol levels one day post-surgery. An analysis of facial expressions revealed a significantly increased SGS on the day of surgery until day 3 post-surgery; this elevated level was sustained until day 17. Clinical severity and SGS scores correlated positively with a Pearson´s correlation coefficient of 0.47. Further investigations regarding the applicability of the SGS revealed a high inter-observer reliability with an intraclass correlation coefficient of 0.92 and an accuracy of 68.2%. In conclusion, the SGS represents a valuable approach for severity assessment that may help support and refine a widely used welfare assessment for sheep during experimental procedures, thereby meeting legislation requirements and minimizing the occurrence of unrecognized distress in animal experimentation.

## Introduction

Directive 2010/63/EU for the protection of animals used for scientific purposes requires an exact severity assessment for all procedures undertaken on laboratory animals. Accordingly, all procedures must be classified into the categories “non-recovery”, “mild”, “moderate” and “severe” on a case-by-case basis (Article 15). Furthermore, a prospective assessment and assignment must be included in applications for the respective project authorization; subsequently, the actual severity of the procedures performed must be documented and reported accordingly (Articles 38, 39 and 54). Severity assessment is also an essential aspect of the 3Rs (reduce, refine, replace) principle [1], which is implemented in the legislative framework (Article 1). Adequate severity assessment requires improved methods to identify disturbed animal welfare. Quantifiable parameters for the classification of severity into the postulated categories remain lacking. Especially, studies investigating standardized tools to quantify experiment severity as well as the animal stress levels are lacking in large animal fracture models (such as tibia osteotomies in sheep). Innovative severity assessment strategies (including objective observations to define the condition of each individual animal) are essential to fulfil the requirements of Directive 2010/63/EU.

Large animals, such as swine or sheep, are often used in biomedical research to study fracture healing and to test new orthopaedic implants [2,3]. For example 28,892 sheep were used in EU member states for experimental and scientific purposes in 2011 [4]. To assess severity in such studies, it is common to apply study-specific scoring sheets based on clinical investigations of the animal’s physiology and behaviour as performed by an experienced veterinarian [5]. However, the obligatory presence of a person may affect the obtained results [6].

In an unilateral tibia osteotomy study in female adult sheep, a suspension system was utilized to prevent postoperative implant failure due to shear stress when lying down or standing up [7]. The system allowed full weight bearing while standing or walking, the latter being ensured by the fixation of the suspension system to a rail. However, during previous in-house studies utilizing a similar suspension system negative side effects such as decubitus with skin necrosis in the axilla were observed (unpublished data). Therefore, the objective of the present study was to assess the severity of the surgical intervention considering the post-operative long-term housing of sheep in a suspension system. Furthermore, it was examined whether the Sheep Grimace Scale (SGS) provide an objective parameter to complement clinical investigations and stress hormone measurements for severity assessment.

Pain assessment by analysing the facial expressions of animals has been reported for different species of laboratory and farm animals. Langford et al. were the first who developed a behavioural coding system based on facial expressions to detect signs of pain in laboratory mice, the Mouse Grimace Scale (MGS) [8]. Following to this, grimace scales for laboratory rats [9] and laboratory rabbits [10] were developed, for domestic cats [11] as well as for farm animals like horses [12], sheep [13] and lambs [14]. Moreover, facial expressions as an indicator for pain are generally applied to assess pain or other emotional states in humans [15]. In the present study post-operative pain and distress in laboratory sheep was analysed and the SGS was established. This was done during the veterinary supervision of an orthopaedic study and was not part of the research project itself. The present study is the first to describe the use of the SGS in assessing post-operative pain and distress in a sheep surgery model.

## Materials and methods

### Animals and housing conditions

**Fig 1:**
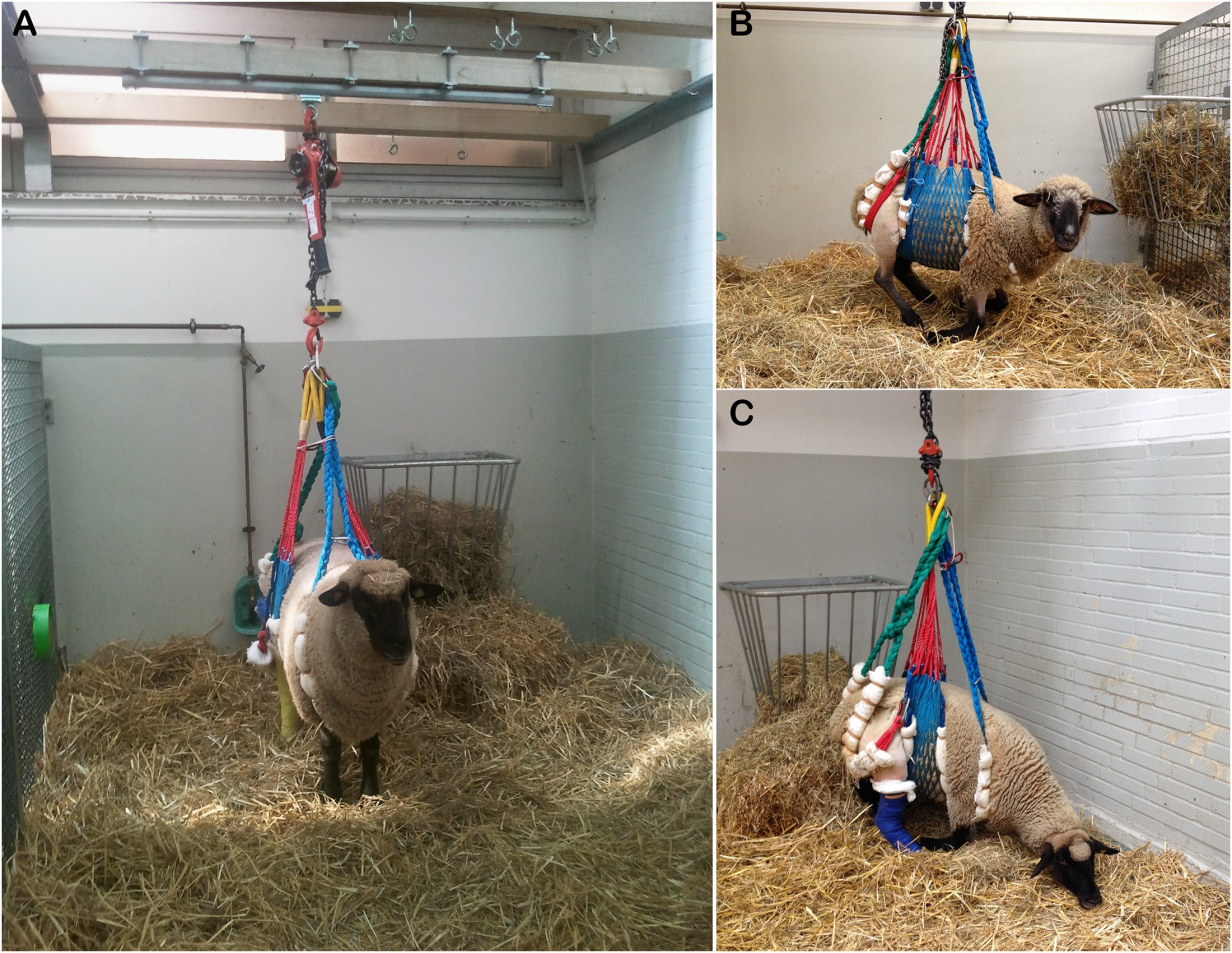
Sheep in a suspension system. The suspension system was loosely wrapped around the rump and prevented the animals from lying down. The system ensured full load bearing while the animals stood and allowed free movement (A), resting (B) and sleeping (C).

A total of 14 female adult blackface sheep aged 3 to 4 years with an average weight of 69.9 kg ± 6.6 kg were obtained from a national breeding farm. Unaffected health status was proofed and verified by a veterinarian on arrival, and the sheep were housed as a flock in an outdoor enclosure. The sheep were fed grain and hay, and water was provided ad libitum. During the experiments, the sheep were singly housed in an indoor enclosure in adjacent cages that allowed eye contact and thus attention to the herd instinct. The sheep were given an adjustment time of 7 days before surgery. To test whether adaptation to the suspension system is beneficial, 5 of the sheep were housed 5 days before surgery in this system. This also enabled an investigation into the impact of this condition without surgery. Postoperatively, the sheep were housed in the suspension system and monitored for 17 consecutive days. The suspension system allowed full weight bearing while standing or walking, resting and sleeping (Fig 1). Fasting was performed for one day prior to surgery. Observation of the animals’ general condition, eating, drinking, defecation, urinating, and gait was performed daily. This study was conducted in accordance with German law for animal protection and with the European Directive, 2010/63/EU. All experiments were approved by the Local Institutional Animal Care and Research Advisory committee and permitted by the local authorities (Lower Saxony State Office for Consumer Protection and Food Safety, LAVES; AZ-12/0967). Severity assessment performed by the animal welfare staff was requested by authorities.

### Surgical procedure and analgesia

Data from this aspect of the study arose from an accompanying surgical study utilizing an unilateral osteotomy model as described elsewhere [7]. Briefly, anaesthesia was introduced via 0.2 mg/kg Midazolam (Dormicum^®^, F. Hoffmann-La Roche AG, Basel, Switzerland) i.v. followed by 2–3 mg/kg Propofol (Propofol-^®^Lipuro 10 mg/ml, B. Braun AG, Melsungen, Germany). After intubation, the right hind legs were shaved, and 4 mg/kg Carprofen (Rimadyl Rind^®^, Pfizer GmbH, Berlin, Germany) were subcutaneously administered. Anaesthesia was maintained by Isoflurane (Isofluran CP^®^, CP-Pharma Handelsgesellschaft mbH, Burgdorf, Deutschland) and 1–10 μg Fentanyl (Fentanyl 0.1 mg, Janssen-Cilag GmbH, Neuss, Germany) to perform tibia osteotomy followed by plate osteosynthesis. At the end of the procedure, 10 μg/kg Buprenorphine (Temgesic^®^, Reckitt Benckiser HealthCare Ltd., Mannheim, Germany) was subcutaneously administered. A cast was applied to stabilize the tibia. Postoperatively, the sheep received 10 μg/kg Buprenorphine twice per day for the first three days and once per day from day 3 to 6 post operation. Additionally, the animals received 2 mg/kg Carprofen subcutaneously for the first 10 days and 1 mg/kg from day 11 to day 13.

Briefly, surgery was performed on the right hind leg. After anaesthesia, the sheep were positioned in the right lateral position. After disinfection and draping, a medial incision for exposure of the right tibia was performed. Following deep dissection, a tibia osteotomy was performed followed by plate osteosynthesis using a new NiTi plate that enables a shape memory effect after transcutaneous induction heating. The wound was then closed in layers, and a sterile draping and cast were applied [7].

### Clinical severity scoring

Each animal was individually assessed according to the clinical severity score presented in Table 1 (adapted from Otto, 2001). Parameters included lameness, posture, rumination, vocalization and general clinical condition. Investigation of the animals was performed by an independent veterinarian at 10:00 AM each day.

**Table 1:** Clinical severity score.

Parameter	Description	Score
Vocalisation (tooth grinding)	none	0
	occasionally	1
	frequently	2
Food/water consumption	normal, rumination	0
	less	1
	none, no rumination	2
Activity	normal resting and sleeping	0
	frequent change of position	1
	restless	2
Lameness	normal standing and walking	0
	normal standing, minimal lameness in exercise	1
	normal standing, lameness in exercise	2
	relief of the affected leg, high lameness in exercise	3
	no usage of the affected leg, predominantly in suspension	4
Maximum score		10

### Saliva sampling and cortisol ELISA

Sheep were given 3 days for habituation after transport to the indoor enclosure. During this time-period the veterinarian responsible for supervision visited the animals and got the animals used to handling procedure. For the collection of saliva animals were restrained by the same veterinarian and sterile cotton swabs were used to collect the specimen. Immediately after sampling, cotton swabs were stored on ice and centrifuged for 10 min at 4500 rpm and 4°C. Samples were stored at -80°C. Salivary cortisol levels were determined via enzyme-linked immunosorbent assay (ELISA) and carried out according to the manufacturer’s standard protocol (Fa. ENZO # ADI-900-071).

### Digital video and editing of footage

A digital video camera was placed on a tripod in front of the cages. Sheep were digitally videotaped using high-resolution (1920 × 1080) digital video cameras (Sony High Definition Handycam^®^ Camcorder; model HDR-CX100) for 30 min one day before surgery (baseline, bsl), 6 to 7 hours after surgery (d0) and on the indicated days post-surgery at 4:00 PM. During videotaping, the researcher left the enclosure to avoid disturbing the animals. Using Microsoft Windows Media Player, clear frames of the videos were selected showing unrestricted views of the face. All suitable pictures per time-point and individual sheep were selected and cropped that only heads were visible.

### Establishment of the SGS

**Fig 2:**
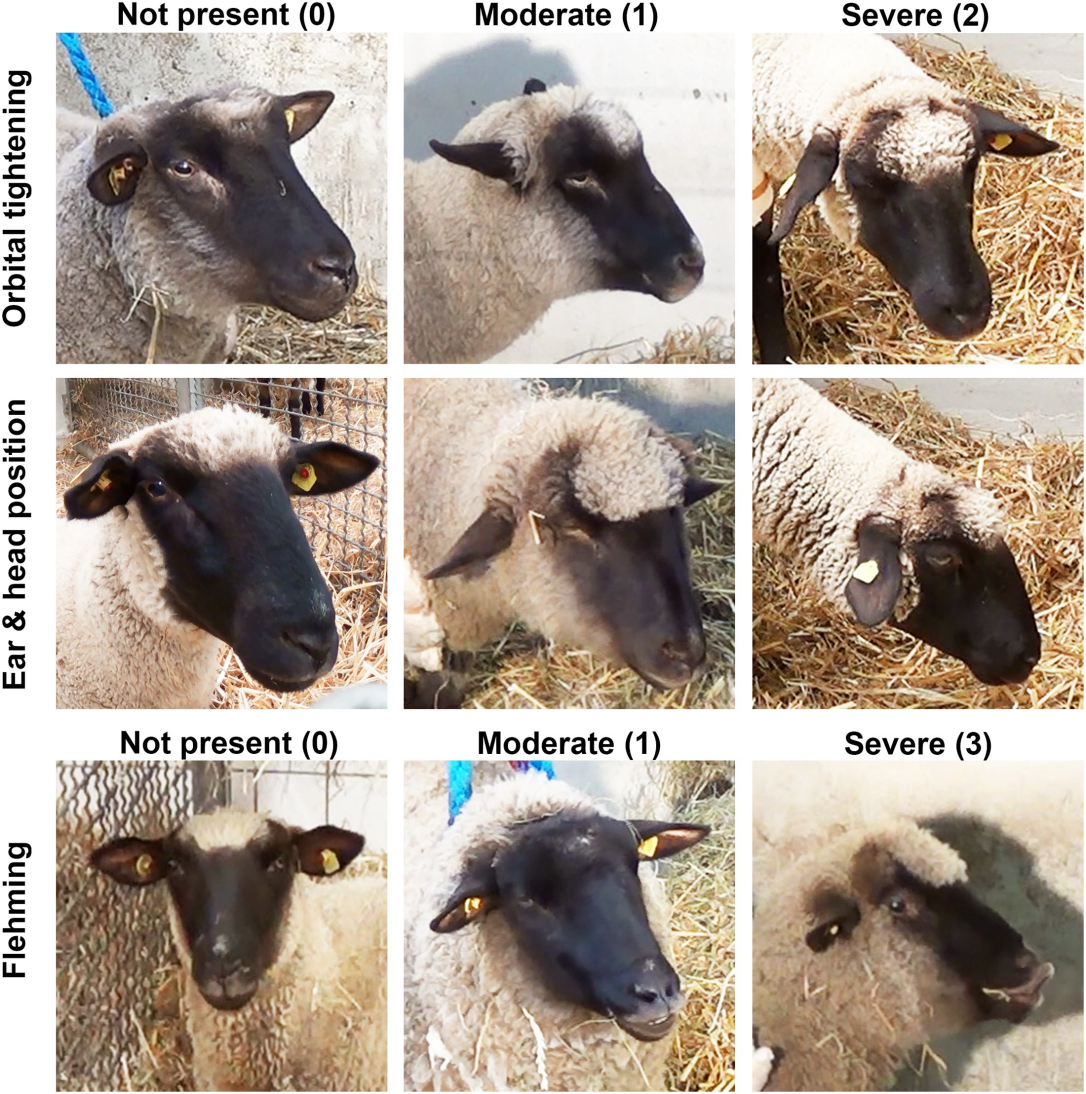
Action units of the Sheep Grimace Scale (SGS).

Pictures from untreated sheep were analysed to classify the status of “pain not present” in which the animals had straightened ears and heads, widely opened eyes and a closed mouth. For the SGS, these action units were scored as 0 (see Fig 2). To further define the grimace scale, pictures were analysed with regard to the pattern of orbital tightening, the position of the ear and head as well as the occurrence of flehming. In orbital tightening, half-closed eyes were defined as an expression of “moderate” pain (score 1), whereas completely closed eyes were assigned with “severe” pain (score 2). Regarding the ear and head position, flattened ears and a slanted head were defined as an expression of moderate pain (score 1), and hanging ears and head were defined as severe (score 2). Flehming represents a sign of severe pain [16]. Due to the fact that sheep lift up their head during flehming, the position of the head cannot be used for evaluation at this time-point and flehmen was therefore matched with a score of 3. In this context, puckered lips were defined as an expression of moderate pain with a score of 1.

A handout containing a collage of photos showing typical examples of each action unit was provided to the scorer and explained by S.B. (who prepared the collage). A group consisting of 5 veterinarians and one scientist, half of them experienced in the detection of postoperative pain in sheep, scored the randomized pictures, while being blinded for time point and treatment. SGS was determined by adding individual scores of facial action units, achieving a maximal possible score of 7.

### Reliability and accuracy determination

Reliability was quantified by comparing the determined SGS scores across the scorer, using the intraclass correlation coefficient, as described elsewhere [8,9]. For the assessment of the accuracy, results from 33 “no pain” pictures (before surgery) and 33 “pain” pictures (post-surgery) were selected and reanalysed in terms of true positives, true negatives, false negatives and false positives by dichotomous judgements.

### Statistics

If not stated otherwise, values are the means ± standard error of the mean. All statistical analyses were performed using GraphPad Prism 5 software (La Jolla, CA). To test distribution of our data sets we performed the Shapiro-Wilk test. For parametric data, one-way repeated measures analysis of variance (ANOVA) was carried out with Bonferroni´s multiple comparisons as a post-hoc test (SGS data). For non-parametric data the Friedman-Test was performed with Dunn´s multiple comparisons as a post-hoc test (clinical severity score data and cortisol data). Strength of correlation was determined by Pearson´s correlation coefficient. The intraclass correlation coefficient (ICC) was calculated using the ICC correlator software (Mangold International GmbH, Germany). P ≤ 0.05 was considered significant. * indicates p ≤ 0.05, ** indicates p ≤ 0.01, and *** indicates p ≤ 0.001.

## Results

### Clinical investigation and stress response analysis

**Fig 3:**
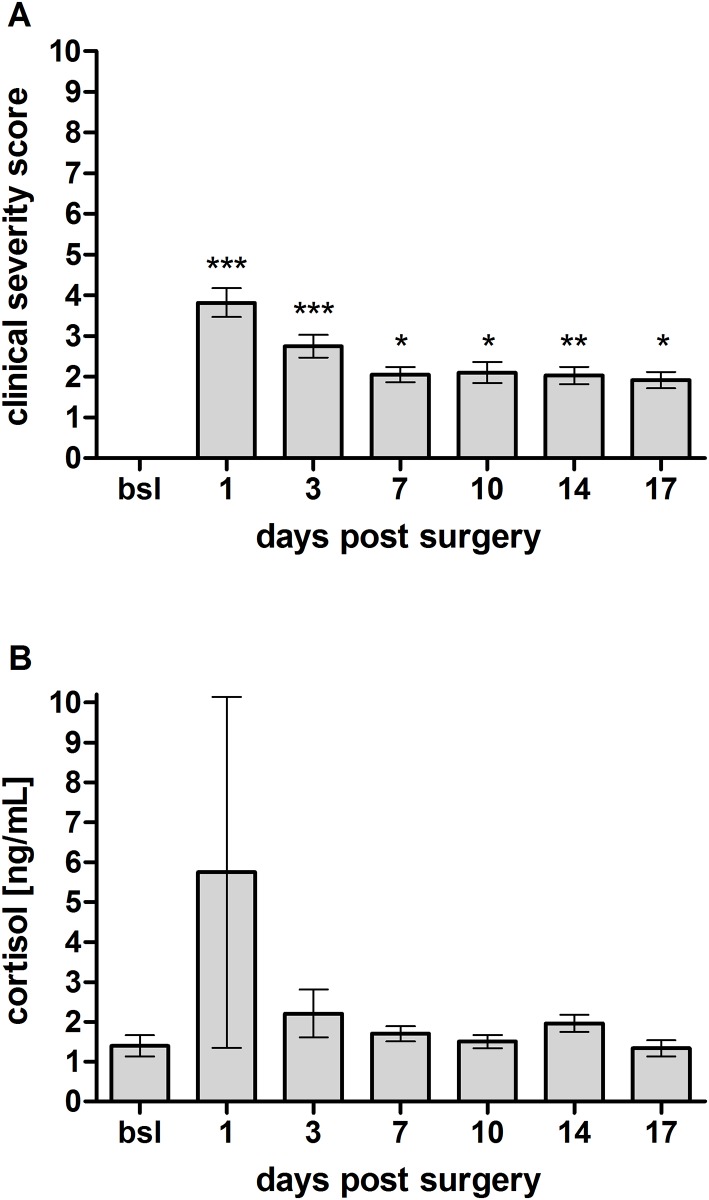
Stress response analysis after unilateral osteotomy. (A) Animals subjected to osteotomy showed significantly elevated clinical severity scores one day post-surgery compared to baseline (bsl) values, which decreased to lower levels but remained elevated until the end of the observation period (n = 14). (B) An analysis of the salivary cortisol level revealed an increase one day post-surgery but a rapid decline to baseline levels on day 3 post surgery (n = 7–8). Significant differences from bsl are indicated by * p ≤ 0.05, ** p ≤ 0.01 and *** p ≤ 0.001.

The clinical investigation was performed using the clinical severity score represented in Table 1. The investigation of sheep 3 days before surgery by a veterinarian resulted in a baseline (bsl) score of 0. The animals undergoing osteotomy showed a significant elevated clinical severity score of 3.8 ± 0.4 (p ≤ 0.001) at day 1 post surgery, which decreased within seven days to a lower level of 2.1 ± 0.2 (p ≤ 0.05). Scores remained at elevated levels compared to bsl until the end of the observation period on day 17 (Fig 3A), mainly due to lameness (data not shown) present to the end of the study in all sheep. Analysis of time as a factor revealed that there was a significant effect of time on clinical severity scoring (p < 0.0001). Assessment of the endocrine stress response after surgery by analysing salivary cortisol levels revealed a statistically not significant increase to 5.8 ± 4.4 ng/ml on day 1 post-surgery compared to the baseline level of 1.4 ± 0.4 ng/ml. In contrast to the continuous presence of clinical signs of distress over the entire observation period, the stress hormone level declined to baseline levels within 7 days (1.7 ± 0.2 ng/ml) post-surgery and remained constant until the end of the observation time (Fig 3B).

**Fig 4:**
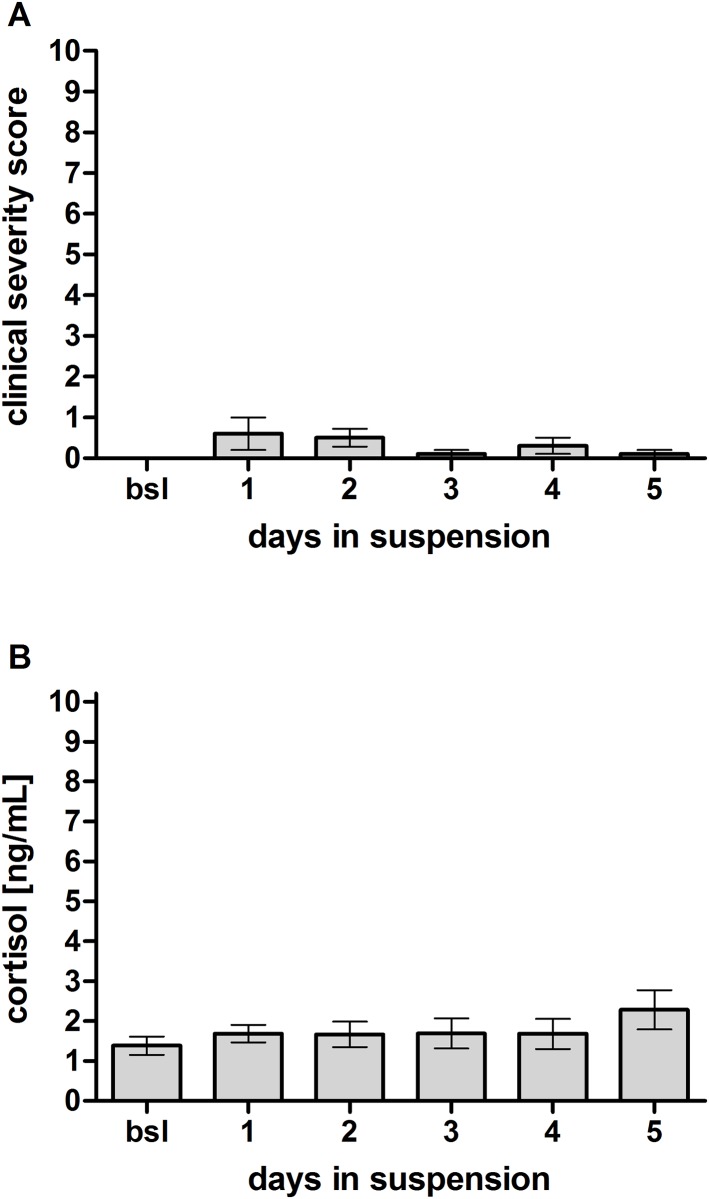
Stress response analysis during maintenance in a suspension system. (A) Clinical severity scoring of untreated animals maintained in a suspension system revealed a slight increase over two days but a reduction until the end of the observation time (n = 5). (B) Analysis of stress hormone response in the saliva of the same animals showed no increase in cortisol levels (n = 5).

To determine whether the suspension system per se puts a strain on the animals during the experimental setup, 5 sheep were mounted into the suspension system for 5 consecutive days prior to surgery. Clinical severity scoring revealed a maximum score of 0.6 ± 0.4 on day 1 after mounting, which decreased to a score of 0.1 ± 0.1 (p > 0.05) on day 5 (Fig 4A). Furthermore, the analysis of salivary cortisol concentrations revealed a slight increase to 1.7 ± 0.2 ng/ml cortisol on day 1 and 2.3 ± 0.5 ng/ml cortisol on day 5 after mounting compared to the baseline cortisol level of 1.4 ± 0.3 ng/ml (p > 0.05) (Fig 4B).

### Severity assessment using the Sheep Grimace Scale

**Fig 5:**
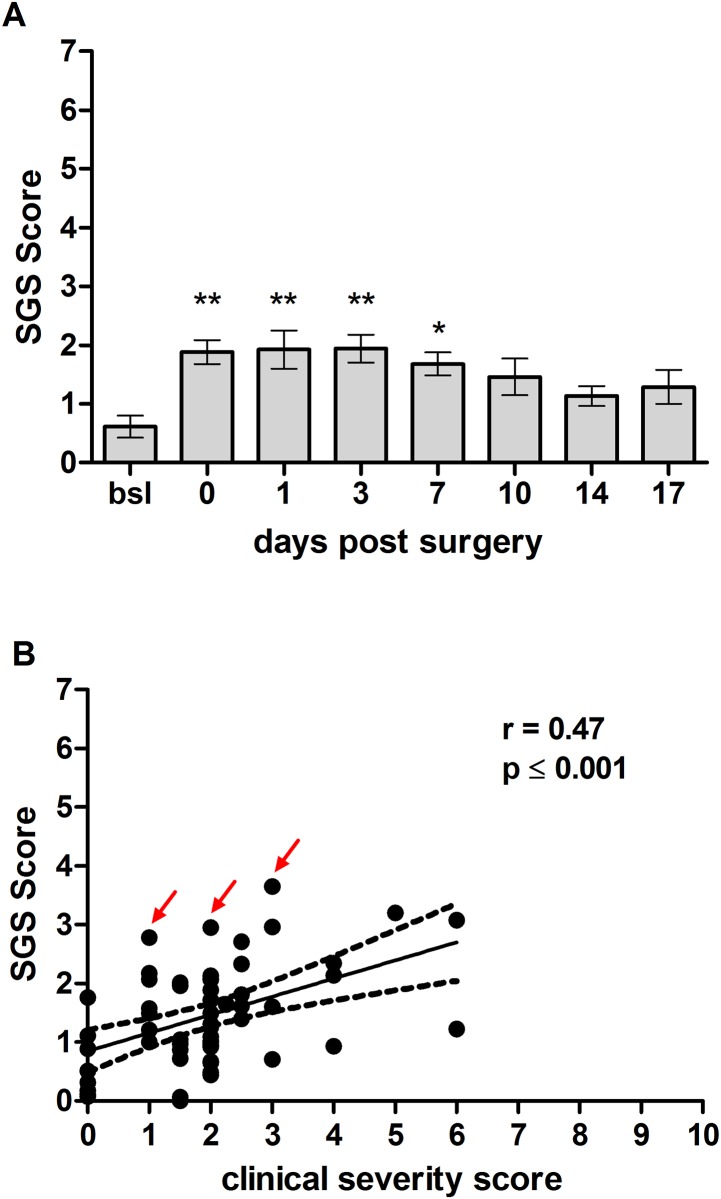
Sheep Grimace Scale after unilateral tibia osteotomy. (A) A few hours post-surgery (day 0), the sheep responded with a SGS score of ~ 1.9, which was significantly increased compared to untreated sheep (bsl), and sustained on this level until day 3 post surgery. The SGS score remained at elevated levels until the end of the observation time (n = 9). (B) shows the significant correlation between clinical severity scores and SGS scores (Pearson´s correlation coefficient 0.47). Relatively high SGS scores compared to clinical severity scores (indicated by red arrows) were identified in sheep with post-operative complications. Significant differences from bsl are indicated by * p ≤ 0.05 and ** p ≤ 0.01.

As shown in Fig 5A, untreated animals (bsl) had a SGS score of 0.6 ± 0.2. Five to six hours post-surgery (indicated as day 0), the sheep responded with a significantly increased SGS score of 1.9 ± 0.2 (p ≤ 0.01), which sustained at this level from day 1 post surgery until day 3 with 1.9 ± 0.2 (p ≤ 0.01), respectively. The SGS score decreased to 1.7 ± 0.2 on day 7 (p ≤ 0.05) and 1.5 ± 0.3 on day 10 (p > 0.05) but remained at elevated levels until day 17 (1.3 ± 0.3) (p > 0.05). Analysis of time as a factor revealed that there was a significant effect of time on sheep grimace scoring (p = 0.004). Comparison of the SGS and the clinical severity score revealed a significant correlation of both scoring methods with a Pearson´s correlation coefficient of 0.47 (p ≤ 0.001) (Fig 5B). Interestingly, values representing extremes above the regression line (indicated by arrows), where the grimace scale was relatively more elevated than the clinical severity score, have been obtained from sheep in which later necropsy revealed implant failures.

### Reliability and accuracy of the Sheep Grimace Scale (SGS)

**Fig 6:**
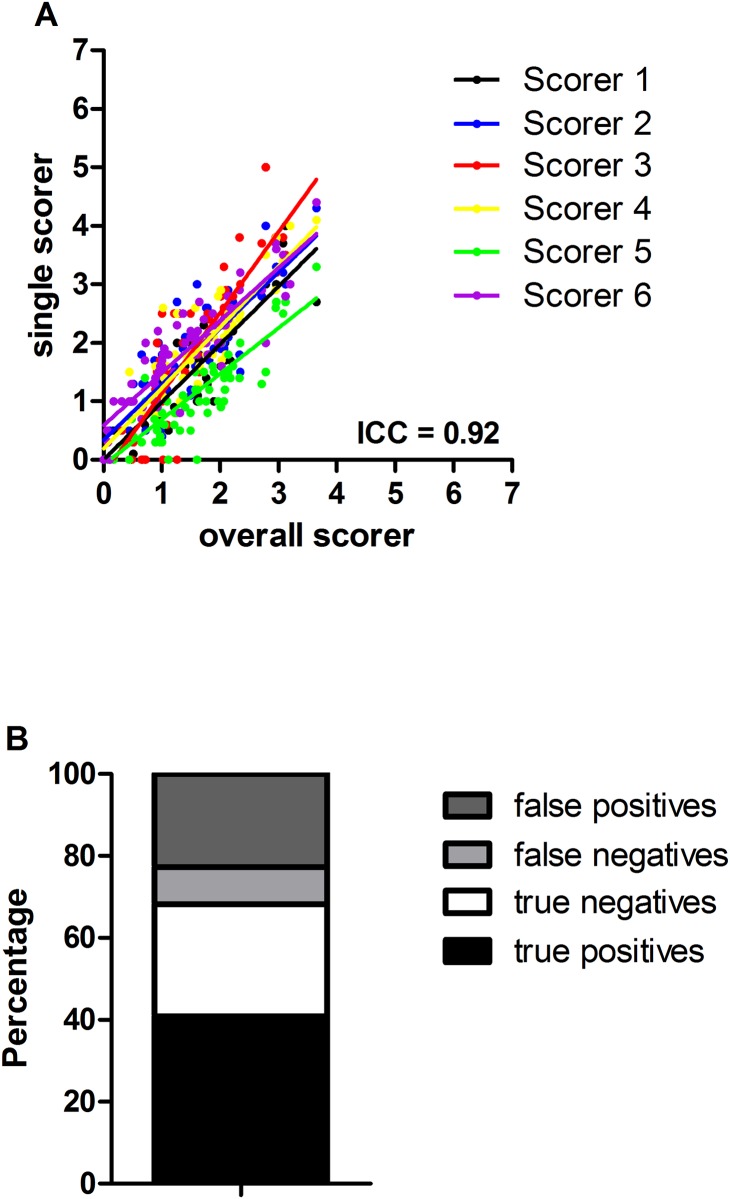
Determination of interrater reliability and accuracy of the SGS. (A) Plotted is the averaged detection data of all scorers (overall) vs data of the single scorer 1–6. Analysis of the interrater reliability revealed an ICC of 0.92. (B) For the determination of the accuracy, 66 pictures were analysed. 33 pictures arising from postoperative sheep and 33 from untreated sheep before surgery were assessed by dichotomous judgement. True positives: pain pictures scored as pain; true negatives: no pain pictures scored as no pain; false negatives: pain pictures scored as no pain; false positives: no pain pictures scored as pain. ICC: intraclass correlation coefficient.

As shown in Fig 6A, analysis of reliability between scorers revealed high inter-rater reliability of the SGS with an ICC of 0.92. Determination of the accuracy revealed a rate of 68.2%, whereas inaccurate determination of pain in “no pain” pictures (false positives: 22.7%) were more common than inaccurate determination of no pain in “pain” pictures (false negatives: 9.1%) (Fig 6B).

## Discussion

Postoperative pain management in large animal models is mandatory for animal welfare and also represents an important factor influencing the outcome of a study. In the present study, for assessment of severity the SGS was established complementary to clinical investigation to test the applicability for severity assessment detecting signs of pain and distress in a sheep surgery model. Furthermore, it was investigated whether a suspension system produces additional distress for the animals following osteotomy.

Clinical investigation and analysis of the hormonal stress response revealed that sheep subjected to an osteotomy and subsequently housed in a suspension system showed an elevated clinical severity score and increased salivary cortisol levels one day post-surgery. The clinical severity score decreased afterwards but was sustained at elevated levels over the entire observation period until day 17, whereas the salivary cortisol levels declined to baseline levels within 7 days post-surgery. Clinical severity scoring and cortisol levels in sheep without surgery but housed on 5 consecutive days in the suspension system were not elevated which indicates that the suspension system was well tolerated by the sheep. Because of this low animal number we can only assume that the clinical severity scores measured post osteotomy provides a measure of the response to surgery rather than to restraint in the suspension system.

Severity assessment in laboratory animals is a complex issue and requires the recognition of pain and stress using a combination of clinical and physiological measurements [17,18]. Animal behaviour represents an important parameter in this assessment. Likewise, the assessment of pain in sheep involves the analysis of behavioural changes such as active pain avoidance behaviour or abnormal posture [19,20]. In particular, pain assessment in lambs that underwent ring castration in the presence of the analgesics flunixin or meloxicam was investigated, showing higher cortisol concentrations in the blood and a preference for taking a pain avoidance posture without anaesthesia [21]. A study investigating the effect of road transport in sheep used various physiological parameters to analyse the stress response and showed hyperthermia and increased cortisol concentrations as well as changes in behaviour such as decreased resting times of the animals [22]. These studies all showed elevated cortisol concentrations; however, the present study showed only a marginal increase of cortisol in saliva. A diminishing factor regarding the endocrine stress response could be the impact of anaesthetic and analgesic drugs. It was shown that cortisol levels increases as a response to castration in lambs and calves. Both studies showed that this increase could be diminished by application of local anaesthesia in combination with analgesia but not under analgesia treatment alone [23,24]. Moreover it has to be considered that glucocorticoids also act in a negative feedback loop and inhibit the production and release of CRF and ACTH and thereby limit both the magnitude and duration of the glucocorticoid increase [25]. Therefore, cortisol as a parameter for stress under chronic stress conditions should be carefully observed because cortisol levels may not correspond with the actual stress experience.

As underlined by the divergent results of clinical scoring and cortisol concentrations after osteotomy, we established the SGS with the intention to develop an improved objective and early detection method for disturbed animal welfare. The analysis of facial expressions in sheep revealed a significantly increased SGS compared to baseline control one day post-surgery. Within 7 days, SGS decreased to a lower but consistently elevated level until day 17, which was similar to the course of the clinical severity score. Interestingly, SGS was relatively more elevated than clinical severity scoring in sheep with disrupted osteosynthesis plates, suggesting that SGS may be more robust in detecting severity than the employed clinical severity score. The utilized SGS revealed a good accuracy (68.2%) with a moderate level of inaccurate determination of pain in “no pain” pictures (false positives: 22.7%), and only a low level of inaccurate determination of no pain in “pain” pictures (false negatives: 9.1%). Compared to other Grimace Scales the accuracy achieved in this study was lower than for the MGS (97%) [8] but similar to the accuracy of the Horse Grimace Scale with 73.3% [12]. The low level of “false negatives” and the more common determination of “false positives” reflect a cautious assessment of pain and might explain elevated SGS in sheep with disrupted osteosynthesis plates.

For the assessment of severity it has to be considered that severity comprises several factors like pain, (emotional) distress, suffering or lasting harm [26]. Determination of animal well-being through clinical investigation was done by scoring of physiological and behavioural parameters. In the clinical severity scoring, lameness was the most characterising factor, but palpation and observation of withdrawing responses did not show any evidence for pain as the causing factor, assuming that the observed lameness might be due to a functional impairment. This raises the question, which dimensions of severity a clinical score assesses and whether this is different to the dimensions of the SGS. As mentioned previously, the interpretation of facial expressions for severity assessment was developed in 2010 by Langford et al. in a surgical mouse model [8], where an elevated MGS score was detected after laparotomy. In a recent study by McLennan et al. the authors developed the sheep pain facial expression scale (SPFES), and showed that on-farm sheep suffering from footrot and mastitis had significantly higher total pain scores on the basis of abnormal facial expressions than control sheep [13]. In comparison to the SGS the SPFES comprises more facial expression areas but matches orbital tightening and ear position, which are despite species-specific differences, common parameters for grimace scaling [27]. In line with the recently published Lamb Grimace Scale (LGS) as an indicator for pain in on-farm lambs after tail docking [14] SPFES and SGS are valid and reliable methods for the detection of pain in sheep. However, it is still unclear whether facial expressions change due to other dimensions of severity like stress or suffering, which are likewise related to pain. In this context, the higher SGS level in sheep with disrupted osteosynthesis plates may be the result of a higher sensitivity; however, further investigations addressing this topic are pivotal.

## Conclusion

The Sheep Grimace Scale is a valuable and reliable method for identifying distress in laboratory sheep. To complement clinical investigation, the SGS represents a potential refinement tool and may help in improving animal welfare conditions under experimentation. The combined severity assessment strategies in this study indicate a moderate distress immediately post-surgery, changing to mild-to-moderate in the following course. Furthermore, the results of the present study indicate that the postoperative utilization of a suspension system following unilateral tibia osteotomy in adult sheep does not present additional distress to the animal in this experimental set up.
